# Depressive and Anxiety Symptoms Among Patients with Asbestos-Related Diseases in Korea

**DOI:** 10.3390/toxics13080703

**Published:** 2025-08-21

**Authors:** Min-Sung Kang, Mee-Ri Lee, Young Hwangbo

**Affiliations:** 1 Asbestos Environmental Health Center, Soonchunhyang University Cheonan Hospital, Soonchunhyang 6-gil 31, Dongnam-gu, Cheonan 31151, Republic of Korea; kms83korea03@hanmail.net; 2 Division of Medical Science, Soonchunhyang University, 22, Soonchunhyang-ro, Sinchang-myeon, Asan 31538, Republic of Korea; 3 Department of Preventive Medicine, College of Medicine, Soonchunhyang University, Soonchunhyang 6-gil 31, Dongnam-gu, Cheonan 31151, Republic of Korea; meeri@sch.ac.kr

**Keywords:** malignant mesothelioma, lung cancer, mental health, depression, anxiety, PHQ-9, GAD-7, HADS

## Abstract

Asbestos-related diseases (ARDs), including malignant mesothelioma, asbestos-related lung cancer, and asbestosis, are known for their long latency periods and poor prognoses. Although the physical effects of ARDs have been widely studied, limited research has examined the psychological burden faced by affected individuals. This study investigated depressive and anxiety symptoms among 275 patients officially recognized as asbestos victims in Korea. Mental health was assessed using the Korean version of the Patient Health Questionnaire-9 (PHQ-9), Generalized Anxiety Disorder-7 (GAD-7), and the Hospital Anxiety and Depression Scale (HADS). The analysis revealed that the mean ± standard deviation of depression and anxiety levels among patients with asbestos-related diseases were 8.06 ± 6.27 for PHQ-9, 6.02 ± 5.64 for GAD-7, 7.09 ± 5.44 for HADS-A, and 8.41 ± 5.47 for HADS-D. Patients with asbestosis had higher levels of depressive and anxiety symptoms than those with malignant mesothelioma or lung cancer, with symptom severity increasing alongside asbestosis grade. When compared with national data from the 2020–2021 Korea National Health and Nutrition Examination Survey (KNHANES), PHQ-9 and GAD-7 scores among ARD patients, particularly those with Grade 1 asbestosis, were higher than the scores reported for all major cancer types. These findings highlight the substantial psychological distress experienced by individuals with ARDs and emphasize the urgent need for targeted mental health interventions in this population.

## 1. Introduction

Asbestos-related diseases, such as malignant mesothelioma, asbestos-related lung cancer, and asbestosis, are specific conditions caused by asbestos exposure and are characterized by a long latency period of approximately 40 years [1]. Although the use of asbestos was comprehensively banned in Korea in 2009, the prolonged latency period has resulted in continued reports of these diseases to the present day. According to data released by the Ministry of Environment, a total of 999 cases of malignant mesothelioma had been reported in Korea as of May 2024, and the number of newly diagnosed patients continues to increase each year.

Asbestos fibers exert their carcinogenic effects through several presumed mechanisms. Once inhaled, fibers can persist in the lungs and pleura due to their biopersistence, leading to chronic inflammation and oxidative stress that promote cellular damage [2]. In addition, asbestos exposure induces the production of reactive oxygen and nitrogen species, which contribute to DNA strand breaks and mutations [3]. Direct physical interaction of fibers with chromosomes during cell division may also cause chromosomal abnormalities [4]. Collectively, these processes create a microenvironment that favors malignant transformation, ultimately resulting in diseases such as mesothelioma and asbestos-related lung cancer [5].

Asbestos-related cancers are generally associated with poor prognoses and extremely low survival rates. In particular, the 5-year survival rate for patients with malignant mesothelioma is reported to be less than 5%, with a median survival time of approximately 15 months [6,7]. A recent study conducted in Korea similarly reported a median survival time of 1.58 years for patients with malignant mesothelioma and 2.58 years for those with asbestos-related lung cancer [8]. Despite significant advances in treatment modalities such as surgery, radiotherapy, and chemotherapy, the survival rate for patients with asbestos-related cancers remains low, highlighting the need for novel complementary approaches [9]. In response, international research has increasingly focused on strategies to identify and manage factors that influence patient survival. Examples include early detection through smoking cessation programs and environmental monitoring systems, as well as efforts to reduce patient exposure to air pollution [8,10,11].

One of the potential factors influencing the survival of patients with asbestos-related diseases is their mental health status. Cancer patients are generally known to experience significant psychological distress during the diagnosis and treatment process, a condition commonly referred to as distress. Numerous prior studies have demonstrated that higher levels of distress are associated with worsened mental health outcomes, which in turn may increase the risk of mortality [12,13]. In particular, the first year following a cancer diagnosis is associated with a marked deterioration in mental health. Compared to the general population, the risk of mental illness among cancer patients has been reported to be approximately 12.6 times higher during the first week after diagnosis and 3.1 times higher within the first year [14]. Accordingly, implementing strategies to support mental health during this critical period may contribute to improvements in both quality of life and life expectancy among cancer patients.

Despite the emphasis in national and international guidelines on the importance of assessing the psychological aspects of patients with asbestos-related diseases [15], few studies have focused directly on the mental health status of these patients [16]. Most existing research has examined the psychological impact on peers, family members, or caregivers of patients [17,18,19,20]. Studies that do focus on patients themselves have predominantly consisted of qualitative investigations, which have provided valuable insights into the subjective experiences of patients with asbestos-related diseases [16,21]. However, despite the important contributions of these qualitative studies, there remains a paucity of research that systematically quantifies psychological distress using standardized instruments such as the Patient Health Questionnaire (PHQ), Generalized Anxiety Disorder (GAD), or Hospital Anxiety and Depression Scale (HADS) [22].

Patients with asbestos-related cancers exhibit several distinct characteristics compared to those with other types of cancer. Notably, the typical survival period of patients with asbestos-related cancers closely overlaps with the time frame during which they are most vulnerable to psychological deterioration. As a result, a sharp decline in quality of life may occur soon after diagnosis, which could directly contribute to reduced survival rates [23,24,25]. Previous studies have also indicated that patients with a greater history of asbestos exposure tend to report higher levels of anxiety [17,22,23,26]. In addition, many patients experience guilt over the possibility that family members may have developed illnesses due to secondary exposure, such as through contaminated work clothing brought home [27,28]. Therefore, implementing intensive mental health support and educational interventions that deliver accurate information immediately following diagnosis may help prevent psychological decline, ultimately contributing to improved quality of life and extended survival among these patients.

Therefore, in order to improve the survival and quality of life of patients with asbestos-related diseases through better mental health management, it is essential to recognize the current challenges and conduct up-to-date research on the psychological impacts of asbestos-related cancers. As a foundational study, the present research aims to investigate the prevalence of depressive and anxiety symptoms among individuals with asbestos-related diseases and to provide evidence supporting the role of mental health management in enhancing patients’ quality of life.

## 2. Materials and Methods

### 2.1. Data Source and Study Population

This study utilized data from patients diagnosed with malignant mesothelioma, asbestos-related lung cancer, or asbestosis, who were officially recognized as asbestos victims under the Asbestos Damage Relief Act in Korea. The identification of asbestos victims followed a structured process administered by the Ministry of Environment.

Individuals who applied for compensation due to asbestos-related diseases were required to submit detailed documentation, including medical records and histories of asbestos exposure, to their respective local governments. In addition, under the National Health Insurance Act, all Korean adults are mandated to undergo regular health screenings. If suspected symptoms of asbestos-related diseases were identified during these examinations, medical institutions were obligated to report the cases to the Ministry of Environment. The Korea Environmental Industry and Technology Institute, an affiliated agency under the Ministry of Environment, reviewed the submitted medical and exposure-related information to assess the causal relationship between asbestos exposure and disease development.

The recognition criteria were strictly defined under the Enforcement Decree of the Asbestos Damage Relief Act. Specifically, malignant mesothelioma was identified through histopathological confirmation or, when not feasible, clinical and radiological evidence. Primary lung cancer required confirmation by pathology or imaging combined with asbestos-related findings such as coexisting asbestosis, pleural plaques, or elevated asbestos body/fiber counts. Asbestosis was determined based on high-resolution CT findings of pulmonary fibrosis categorized as suspected, early, or progressive, in combination with pulmonary function impairment levels. Furthermore, asbestosis was graded according to both radiological severity and functional impairment: Grade 1 required either (i) early or progressive asbestosis with severe functional impairment, or (ii) progressive asbestosis with moderate impairment; Grade 2 applied to progressive asbestosis without functional impairment or early asbestosis with moderate impairment; and Grade 3 applied to early asbestosis without functional impairment, provided that latency and exposure history supported causal inference.

Based on this comprehensive evaluation, individuals were officially classified as recognized victims of asbestos exposure [1,8,29]. Subsequently, trained specialists from the Environmental Health Center for Asbestos at Soonchunhyang University Cheonan Hospital conducted field visits to these confirmed victims. During these visits, data were collected through structured interviews that covered basic demographic information as well as self-reported mental health status, including depressive and anxiety symptoms. In 2024, a total of 275 patients classified as having asbestos-related diseases were surveyed, including 4 patients with malignant mesothelioma, 21 with asbestos-related lung cancer, and 250 with asbestosis. Because the study targeted the entire accessible population of patients officially recognized as asbestos victims during the study period, a priori statistical power calculation was not applicable. Instead, the study included all eligible individuals, which ensured that the sample size reflected the full scope of available cases of asbestos-related diseases in Korea.

The institutional review board of Soonchunhyang University Cheonan Hospital approved the collection and utilization of data for this study (2024-03-030).

### 2.2. Assessment of Depression and Anxiety

To evaluate mental health status among participants, symptoms of depression and anxiety were assessed using three standardized self-report instruments: the Patient Health Questionnaire-9 (PHQ-9), the Generalized Anxiety Disorder-7 (GAD-7), and the Hospital Anxiety and Depression Scale (HADS), Korean version.

Depressive symptoms were initially measured using the PHQ-9, a brief screening tool developed to assess the frequency of nine core symptoms associated with major depressive disorder over the past two weeks. Each item is rated on a four-point Likert scale ranging from 0 (“not at all”) to 3 (“nearly every day”), resulting in a total score between 0 and 27. According to established guidelines, total scores of 5–9 are considered indicative of mild depressive symptoms, 10–14 suggest moderate severity, and scores of 15 or above denote severe depressive symptoms. The PHQ-9 has demonstrated strong psychometric properties across various demographic groups [30].

Anxiety symptoms were assessed using the GAD-7, which consists of seven items reflecting the frequency of general anxiety-related experiences within the past two weeks. Each item is scored on a scale from 0 (“not at all”) to 3 (“nearly every day”), producing total scores that range from 0 to 21. Higher scores are associated with greater symptom burden, with interpretive cutoffs commonly set at 5 (mild), 10 (moderate), and 15 (severe) anxiety. The GAD-7 is a widely validated tool for identifying probable cases of generalized anxiety disorder [31].

In addition to these scales, the Korean version of the Hospital Anxiety and Depression Scale (HADS) was administered to further characterize participants’ emotional distress. Designed to screen for the presence and severity of anxiety and depressive symptoms in both clinical and community settings, the HADS includes 14 items divided equally into two subscales: HADS-A for anxiety and HADS-D for depression [32,33]. Each item is scored on a 4-point scale (0–3), and total subscale scores range from 0 to 21. Scores between 8 and 10 suggest the possible presence of anxiety or depression, while a score of 11 or above indicates probable symptoms of the respective condition [34]. Unlike diagnostic tools, the HADS focuses on symptom intensity rather than clinical categorization, making it suitable for use in populations with physical illnesses. Its validity and reliability have been confirmed in numerous international and Korean studies, particularly for use in medically ill populations [35].

### 2.3. Statistical Analysis

We conducted univariate analyses to examine depression and anxiety scores according to participant characteristics. Means were presented as point estimates, and variability in scores was assessed using standard deviations (SD). Differences in mean scores were evaluated using a *t*-test or analysis of variance (ANOVA). The prevalence of depressive and anxiety symptoms was calculated for each scale based on established subscale thresholds. For PHQ-9 and GAD-7, symptom severity was categorized as mild (scores of 5–9), moderate (10–14), and severe (15 or higher). For the HADS, scores of 8–10 were classified as suggestive of anxiety or depression, while scores of 11 or higher were considered indicative of probable cases. The statistical significance of differences in prevalence across disease types was assessed using the chi-square test.

To adjust for the influence of covariates on depression and anxiety, adjusted mean depression and anxiety scores by disease type were estimated using analysis of covariance (ANCOVA). Covariates included sex (categorical), age (continuous), smoking status (categorical), and residential area (categorical).

### 2.4. Comparison with Other Cancers

To compare the depression and anxiety scores of individuals with asbestos-related diseases to those of patients with other types of cancer, we used data from the Korea National Health and Nutrition Examination Survey (KNHANES), which is representative of the general Korean population. Mean PHQ-9 and GAD-7 scores for cancer patients were calculated based on the 2020–2021 KNHANES data, as the 2022–2024 data did not include information on cancer diagnosis.

## 3. Results

The general characteristics of participants are presented in Table 1. Across malignant mesothelioma, asbestos-related lung cancer, and asbestosis, male patients outnumbered female patients. Most patients were aged 70–89 years, and geographically, cases were concentrated in Busan and South Chungcheong Province (Chungcheongnam-do).

Table 2 presents depression and anxiety scores according to participants’ demographic characteristics. For smoking status, a trend was observed in which higher frequency of smoking was associated with higher scores across all measures, except for HADS-A. Regarding disease type, patients with asbestosis showed higher depression and anxiety scores compared to those with malignant mesothelioma or lung cancer. In addition, among asbestosis patients, there was a tendency for scores to increase with the severity of the condition.

Table 3 presents the number and percentage of patients within each subscale category of PHQ-9 and GAD-7. Because the number of malignant mesothelioma patients (*n* = 4) was too small for meaningful statistical comparison, we did not compare this group directly with other diagnoses. When focusing on asbestos-related lung cancer and asbestosis, the prevalence of depression and anxiety symptoms was higher among patients with asbestosis. Moreover, within the asbestosis group, there was a clear trend showing that the proportion of individuals scoring in the moderate or higher range (≥10 points) increased with disease severity.

Table 4 shows the distribution of patients by HADS-A (anxiety) and HADS-D (depression) subscales. Due to the very small number of malignant mesothelioma patients, no direct comparison with other disease groups was made. When comparing asbestos-related lung cancer and asbestosis, the prevalence of depression (HADS-D) was higher among patients with asbestosis, while no significant difference was observed for anxiety (HADS-A). In addition, among asbestosis patients, the proportion of individuals scoring in the probable range (≥11 points) tended to increase with greater disease severity.

Table 5 presents adjusted mean scores of depression and anxiety after controlling for sex, age, smoking status, and residential area. Because of the small sample size, the malignant mesothelioma group was not compared with other groups. In comparisons between asbestos-related lung cancer and asbestosis, patients with asbestosis generally exhibited higher adjusted scores for both depression and anxiety measures (PHQ-9, GAD-7, HADS-A, HADS-D). Consistent with the unadjusted results, mean scores increased in parallel with the severity of asbestosis, reinforcing the dose–response relationship between disease progression and psychological distress. The results of the regression analysis examining the associations between demographic variables other than the type of asbestos-related disease and depression and anxiety scores are presented in Table S1.

**Figure 1:**
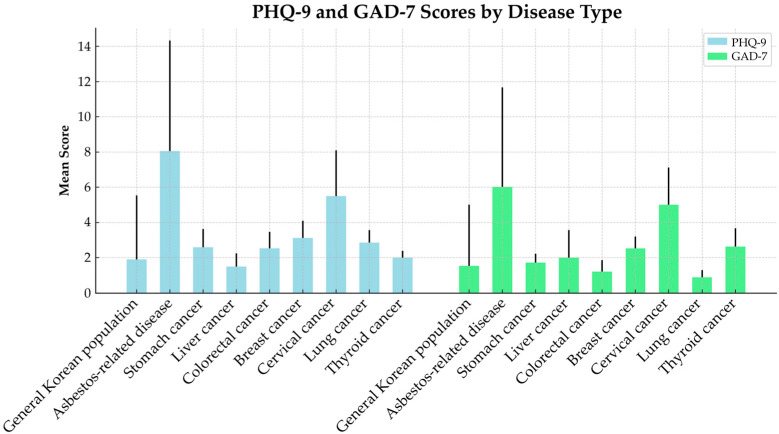
Mean and standard deviation of PHQ-9 and GAD-7 scores by disease type. The mean scores for cancers other than asbestos-related disease were derived using data from the 2020 and 2021 Korea National Health and Nutrition Examination Survey (KNHANES). Blue and green bars represent the mean scores of PHQ-9 and GAD-7, and black lines indicate standard deviation.

Figure 1 presents a comparison of PHQ-9 and GAD-7 scores between patients with asbestos-related diseases in this study, patients with major cancer types in Korea, and the general Korean population, based on data from the 2020–2021 KNHANES. For the depression scale (PHQ-9), the mean scores were as follows: stomach cancer 2.60, liver cancer 1.50, colorectal cancer 2.53, breast cancer 3.11, cervical cancer 5.50, lung cancer 2.86, and thyroid cancer 2.00, while the general population scored 1.91. For the anxiety scale (GAD-7), the mean scores were as follows: stomach cancer 1.71, liver cancer 2.00, colorectal cancer 1.21, breast cancer 2.53, cervical cancer 5.00, lung cancer 0.89, and thyroid cancer 2.63, with the general population scoring 1.53. The PHQ-9 scores observed among patients with asbestos-related diseases in this study were higher than those reported for all cancer types in the KNHANES data and were approximately four times higher compared with the general Korean population. Similarly, the GAD-7 scores among patients with asbestos-related diseases exceeded those of all cancer patients surveyed in KNHANES and were also about four times higher compared with the general population. Notably, the PHQ-9 score for patients with Grade 1 asbestosis was more than four times higher than the scores reported for all cancer types in the KNHANES data, and a similar pattern was observed for GAD-7 scores. In particular, the PHQ-9 score for Grade 1 asbestosis patients was more than twice as high as that of cervical cancer patients, who had the highest mean score among all cancer types in KNHANES, indicating a markedly elevated level of depressive symptoms among individuals with asbestosis.

## 4. Discussion

The aim of this study was to examine the levels of depression and anxiety among patients in Korea diagnosed with malignant mesothelioma, asbestos-related lung cancer, and asbestosis. Mental health data from a total of 275 patients, collected by the Ministry of Environment and the Environmental Health Center for Asbestos, were analyzed. Each patient’s level of depression and anxiety was quantified using standardized instruments, including the PHQ-9, GAD-7, and HADS. Patients with asbestosis reported higher levels of depressive and anxiety symptoms compared to those with malignant mesothelioma or lung cancer, with symptom severity increasing according to the grade of asbestosis. When compared to data from the 2020–2021 KNHANES, PHQ-9 and GAD-7 scores among patients, particularly those with Grade 1 asbestosis, were higher than the scores reported for all major cancer types. Moreover, compared with the general Korean population (PHQ-9 = 1.91, GAD-7 = 1.53), the mean scores of patients with asbestos-related diseases were approximately four times higher, underscoring the particularly severe psychological burden experienced by this group.

There are very few studies that have quantitatively assessed the mental health of patients with asbestos-related diseases using instruments such as the PHQ-9, GAD-7, or HADS. To our knowledge, only two studies have used the HADS to quantify levels of depression and anxiety in patients with malignant mesothelioma. Sherborne et al. measured depression and anxiety in a total of 96 individuals in the UK, including adults aged 18 and older who were either diagnosed with malignant mesothelioma or serving as caregivers for such patients [36]. The results showed that the mean score on the HADS-A was 7.91 (SD = 4.18), and the mean score on the HADS-D was 5.83 (SD = 4.12). Additionally, Mounchetrou Njoya et al. assessed levels of depression and anxiety among 2225 voluntary participants undergoing follow-up CT scans from the French Asbestos-Related Diseases Cohort (ARDCO) [22]. They reported a mean HADS-A score of 7.26 (SD = 3.99) and a mean HADS-D score of 5.53 (SD = 3.77). These values are comparable to the mean HADS-A and HADS-D scores observed among malignant mesothelioma patients in our study. The levels of depression and anxiety observed in both previous studies and the present study among patients with asbestos-related diseases are significantly higher than those reported for other cancer patients based on KNHANES data, and this trend is supported by findings from other research as well. Three studies that assessed depression in cancer patients using the PHQ-9 reported mean PHQ-9 scores of 5.14, 5.26, and 6.5, respectively [37,38,39]. Additionally, a study that measured anxiety in cancer patients using the GAD-7 reported a mean GAD-7 score of 3.89, which are significantly lower than the means reported in our study [37].

The poorer mental health observed among individuals with asbestos-related diseases may be associated with the unique psychological characteristics and psychosocial burdens specific to this population. Evidence from national survey data (KNHANES) suggests that cancer patients, in general, report comparatively lower levels of psychological distress than patients with asbestos-related diseases, further underscoring the distinctive challenges faced by individuals with asbestos-related diseases. This pattern was not only evident when compared to cancer patients but also when contrasted with the general Korean population, where average PHQ-9 and GAD-7 scores were substantially lower. Such results highlight that individuals with asbestos-related diseases experience disproportionately higher levels of depression and anxiety than both cancer patients and the general public. In general, cancer patients tend to experience a decline in mental health due to the suffering caused by the illness itself and the challenges associated with treatment [12,13], which differs from mental health deterioration triggered by factors such as exposure to neuroendocrine-disrupting chemicals [40,41,42,43], dietary intake [44,45,46], or trauma-inducing disasters [47,48,49,50]. Additionally, patients with asbestos-related diseases are characterized by an increased awareness of the dangers of asbestos and the severity of asbestos-related illnesses [25]. A systematic review by Bonafede et al. on the psychological distress of patients with malignant mesothelioma and asbestos-exposed individuals provides insight into these characteristics [16]. Such individuals often display heightened sensitivity to the perceived inevitability of severe illness following asbestos exposure [25], harbor anger toward employers they believe responsible for their exposure [24], and experience guilt stemming from concerns about familial contamination [28]. In particular, patients with asbestosis frequently report fear that symptoms of malignant mesothelioma or lung cancer may develop in the future [51]. In our study, we also observed that depressive and anxiety symptoms became more pronounced as the severity of asbestosis increased, suggesting that such fears of future health deterioration may exacerbate mental health problems in this population. Another important factor is survival duration: unlike patients with malignant mesothelioma or lung cancer, who often face a relatively short survival time following diagnosis, individuals with asbestosis experience a gradual progression of symptoms and therefore live with the disease for a longer period. This prolonged survival may increase the likelihood of enduring psychological distress over time. Similar patterns have been documented in patients with chronic obstructive pulmonary disease (COPD), where the chronic and progressive nature of the illness leads to functional disability, dyspnea, reduced quality of life, and subsequent depression and anxiety [52,53]. These characteristics may help explain why, in our study, patients with asbestosis demonstrated higher levels of depression and anxiety compared to those with malignant mesothelioma or lung cancer, and why their mental health declined further with increasing disease severity.

Several previous studies have reported that support groups for individuals with asbestos-related diseases have a positive impact on their mental health [24]. For instance, the UK-based program Hands of Time provided lectures for patients with malignant mesothelioma and their families on topics such as treatment options, emerging therapies, compensation claims, breathing and exercise techniques, dietary management, and the importance of maintaining a positive mindset [54]. These activities facilitated information sharing and emotional bonding among participants. Similar initiatives are being implemented by Korea’s environmental health center for asbestos [29]. The center aims to enhance awareness, emotional well-being, and education for both asbestos-affected individuals and the broader community. Their programs include eco-tours and recreational sessions, such as singing and laughter activities, that promote relaxation and social connection. In addition, targeted presentations on legal systems and support services for asbestos victims are delivered to healthcare providers, and a variety of informational resources, including leaflets, booklets, and periodicals, are routinely distributed to both victims and the general public. Given that the levels of depression and anxiety observed in our study were markedly higher not only than those of other cancer patients but also nearly four times greater than those of the general Korean population, such support activities should be further expanded and tailored to meet the urgent psychosocial needs of asbestos-related disease patients.

This study has several limitations that should be acknowledged. First, the number of patients with malignant mesothelioma was relatively small, which limited the reliability of comparisons with other patient groups. However, malignant mesothelioma is a rare disease, and the one-year study period restricted our ability to recruit more participants. Nevertheless, the total number of asbestos-related disease patients included (*n* = 275) was comparable to previous studies, and it was sufficient for comparisons with cancer patients identified in national survey data (KNHANES). Second, information on individual socioeconomic status, such as education and income level, was not incorporated into the analyses. Although these variables were initially included in the survey, many respondents were reluctant to disclose such information, and the data were therefore excluded. To address this issue, in our previous study we conducted analyses adjusted by quartiles of the average education and income levels across administrative regions. However, no significant changes were observed in the original results [8]. It is likely because many of the patients were concentrated in regions with established asbestos exposure sources, leading to relatively little variation in regional socioeconomic characteristics. Finally, the cross-sectional design of this study precludes the establishment of causal relationships between asbestos-related diseases and mental health outcomes. Longitudinal studies are needed to clarify the temporal dynamics between disease progression and psychological distress.

## 5. Conclusions

In this study, we investigated depressive and anxiety symptoms among patients officially recognized as asbestos victims in Korea. Based on the findings of this study, individuals with asbestos-related diseases, particularly those with asbestosis, experience significant psychological distress, often exceeding that observed in patients with other major cancer types. These results suggest that current care strategies for asbestos-related disease patients should incorporate comprehensive psychosocial interventions.

## Figures and Tables

**Table 1:** Characteristics of study population with asbestos-related diseases.

Variables	Malignant Mesothelioma	Lung Cancer	Asbestosis		
			Grade 1: Severe	Grade 2: Moderate	Grade 3: Mild
Total	4 (100.0)	21 (100.0)	16 (100.0)	95 (100.0)	139 (100.0)
Sex					
Male	2 (50.0)	16 (76.2)	13 (81.3)	65 (68.4)	67 (48.2)
Female	2 (50.0)	5 (23.8)	3 (18.8)	30 (31.6)	72 (51.8)
Age					
50–59	0 (0.0)	0 (0.0)	0 (0.0)	3 (3.2)	1 (0.7)
60–69	3 (75.0)	6 (28.6)	0 (0.0)	11 (11.6)	22 (15.8)
70–79	1 (25.0)	10 (47.6)	9 (56.3)	36 (37.9)	56 (40.3)
80–89	0 (0.0)	5 (23.8)	7 (43.8)	39 (41.1)	53 (38.1)
≥90	0 (0.0)	0 (0.0)	0 (0.0)	6 (6.3)	7 (5.0)
Smoking status					
Never	0 (0.0)	3 (14.3)	1 (6.3)	18 (18.9)	62 (44.6)
Past smoker	0 (0.0)	12 (57.1)	7 (43.8)	19 (20.0)	23 (16.5)
Current smoker	0 (0.0)	0 (0.0)	0 (0.0)	3 (3.2)	5 (3.6)
Unknown	4 (100.0)	6 (28.6)	8 (50.0)	55 (57.9)	49 (35.3)
Region					
Seoul	4 (100.0)	0 (0.0)	0 (0.0)	1 (1.1)	1 (0.7)
Incheon	0 (0.0)	1 (4.8)	0 (0.0)	0 (0.0)	6 (4.3)
Gyeonggi-do	0 (0.0)	1 (4.8)	3 (18.8)	5 (5.3)	8 (5.8)
Gyeonsangnam-do	0 (0.0)	4 (19.0)	0 (0.0)	4 (4.2)	1 (0.7)
Busan	0 (0.0)	5 (23.8)	2 (12.5)	25 (26.3)	21 (15.1)
Chungcheongnam-do	0 (0.0)	10 (47.6)	11 (68.8)	60 (63.2)	102 (73.4)

**Table 2:** Mean and standard deviation of depression and anxiety scores by characteristics of patients.

Variables	*n* (%)	PHQ-9		GAD-7		HADS-A		HADS-D	
		Mean ± SD	*p*	Mean ± SD	*p*	Mean ± SD	*p*	Mean ± SD	*p*
Total	275 (100.0)	8.06 ± 6.27		6.02 ± 5.64		7.09 ± 5.44		8.41 ± 5.07	
Sex									
Male	163 (59.3)	8.23 ± 6.25	0.599	6.02 ± 5.56	0.997	7.28 ± 5.49	0.496	8.73 ± 4.99	0.209
Female	112 (40.7)	7.82 ± 6.32		6.02 ± 5.79		6.82 ± 5.39		7.95 ± 5.18	
Age									
50–59	4 (1.5)	4.50 ± 2.08	0.623	3.25 ± 3.95	0.647	3.75 ± 4.50	0.122	4.00 ± 2.94	0.007
60–69	45 (15.3)	7.21 ± 6.00		5.33 ± 5.66		5.57 ± 5.20		6.55 ± 4.92	
70–79	112 (40.7)	8.07 ± 6.32		5.96 ± 5.37		7.21 ± 5.14		8.64 ± 4.63	
80–89	104 (37.8)	8.53 ± 6.61		6.33 ± 6.05		7.43 ± 5.82		8.71 ± 5.42	
≥90	13 (4.7)	8.08 ± 4.44		7.23 ± 5.15		9.23 ± 5.04		11.38 ± 4.72	
Smoking status									
Never	185 (67.3)	6.73 ± 6.15	0.005	4.67 ± 5.61	0.008	5.63 ± 5.38	0.001	7.37 ± 5.53	0.034
Past smoker	61 (22.2)	7.00 ± 5.38		5.44 ± 5.16		6.44 ± 5.77		7.87 ± 4.77	
Current smoker	8 (2.9)	7.38 ± 5.83		5.38 ± 4.50		5.63 ± 3.16		9.00 ± 3.38	
Unknown	21 (7.6)	9.56 ± 6.53		7.30 ± 5.75		8.52 ± 5.12		9.36 ± 4.85	
Region									
Seoul	6 (2.2)	7.50 ± 6.89	0.225	4.50 ± 5.96	0.113	6.67 ± 3.83	0.009	6.17 ± 4.62	0.222
Incheon	7 (2.5)	7.71 ± 4.11		5.57 ± 5.03		8.57 ± 5.06		6.57 ± 3.74	
Gyeonggi-do	17 (6.2)	8.24 ± 4.63		5.00 ± 4.02		6.65 ± 3.95		7.82 ± 3.63	
Gyeonsangnam-do	9 (3.3)	3.33 ± 4.50		2.89 ± 4.73		3.22 ± 6.02		5.56 ± 5.90	
Busan	53 (19.3)	9.19 ± 5.98		7.74 ± 5.51		9.28 ± 5.42		9.34 ± 4.46	
Chungcheongnam-do	183 (66.5)	7.98 ± 6.54		5.85 ± 5.79		6.64 ± 5.43		8.48 ± 5.32	
Diagnosis									
Malignant mesothelioma	4 (1.5)	6.50 ± 7.42	0.005	3.75 ± 7.50	0.024	6.50 ± 4.66	0.017	5.50 ± 5.80	0.007
Lung cancer	21 (7.6)	6.62 ± 6.00		4.71 ± 5.82		6.33 ± 7.10		6.38 ± 4.09	
Asbestosis (Grade 1: Severe)	16 (5.8)	11.75 ± 4.92		8.88 ± 5.18		9.88 ± 4.88		10.75 ± 3.28	
Asbestosis (Grade 2: Moderate)	95 (34.5)	9.36 ± 6.43		7.01 ± 5.62		8.13 ± 5.29		9.44 ± 4.94	
Asbestosis (Grade 3: Mild)	139 (50.6)	7.01 ± 6.05		5.29 ± 5.49		6.19 ± 5.18		7.83 ± 5.25	

**Table 3:** Association between asbestos-related diseases and subscale scores of PHQ-9 and GAD-7.

Variables	PHQ-9 Subscale Score [*n* (%)]				GAD-7 Subscale Score [*n* (%)]			
	Mild (5–9)	Moderate (10–14)	Severe (≥15)	*p*	Mild (5–9)	Moderate (10–14)	Severe (≥15)	*p*
Total (*n* = 275)	67 (24.4)	63 (22.9)	44 (16.0)		83 (30.2)	41 (14.9)	29 (10.5)	
Diagnosis								
Malignant mesothelioma (*n* = 4)	1 (25.0)	0 (0.0)	1 (25.0)	0.057	0 (0.0)	0 (0.0)	1 (25.0)	0.008
Lung cancer (*n* = 21)	4 (19.0)	4 (19.0)	3 (14.3)		5 (23.8)	1 (4.8)	2 (9.5)	
Asbestosis (Grade 1: Severe) (*n* = 16)	3 (18.8)	9 (56.3)	3 (18.8)		5 (31.3)	7 (43.8)	1 (6.3)	
Asbestosis (Grade 2: Moderate) (*n* = 95)	24 (25.3)	24 (25.3)	19 (20.0)		32 (33.7)	20 (21.1)	10 (10.5)	
Asbestosis (Grade 3: Mild) (*n* = 139)	35 (25.2)	26 (18.7)	18 (12.9)		41 (29.5)	13 (9.4)	15 (10.8)	

**Table 4:** Association between asbestos-related diseases and subscale scores of HADS-A and HADS-D.

Variables	HADS-A Subscale Score [*n* (%)]			HADS-D Subscale Score [*n* (%)]		
	Suggestive (8–10)	Probable (≥11)	*p*	Suggestive (8–10)	Probable (≥11)	*p*
Total (*n* = 275)	48 (17.5)	71 (25.8)		61 (22.2)	90 (32.7)	
Diagnosis						
Malignant mesothelioma (*n* = 4)	1 (25.0)	1 (25.0)	0.256	0 (0.0)	1 (25.0)	0.010
Lung cancer (*n* = 21)	1 (4.8)	5 (23.8)		3 (14.3)	4 (19.0)	
Asbestosis (Grade 1: Severe) (*n* = 16)	2 (12.5)	7 (43.8)		3 (18.8)	10 (62.5)	
Asbestosis (Grade 2: Moderate) (*n* = 95)	21 (22.1)	28 (29.5)		29 (30.5)	33 (34.7)	
Asbestosis (Grade 3: Mild) (*n* = 139)	23 (16.5)	30 (21.6)		26 (18.7)	42 (30.2)	

**Table 5:** Adjusted ^1^ depression and anxiety scores according to diagnosis of patients.

Variables	PHQ-9		GAD-7		HADS-A		HADS-D	
	β (95% CI)	*p*	β (95% CI)	*p*	β (95% CI)	*p*	β (95% CI)	*p*
Diagnosis								
Malignant mesothelioma	4.47 (−4.99, 13.92)	0.029	3.35 (−5.20, 11.99)	0.111	6.43 (−1.68, 14.53)	0.042	6.35 (−1.28, 13.98)	0.030
Lung cancer	7.09 (3.71, 10.47)		4.69 (1.63, 7.75)		6.69 (3.79, 9.59)		6.55 (3.83, 9.28)	
Asbestosis (Grade 1: Severe)	10.87 (7.07, 14.67)		8.31 (4.87, 11.75)		9.47 (6.21, 12.73)		10.01 (6.94, 13.08)	
Asbestosis (Grade 2: Moderate)	8.34 (5.89, 10.79)		6.13 (3.91, 8.35)		7.43 (5.33, 9.53)		8.53 (6.55, 10.51)	
Asbestosis (Grade 3: Mild)	6.29 (3.99, 8.60)		4.78 (2.70, 6.87)		5.87 (3.90, 7.85)		7.37 (5.51, 9.23)	

^1^ All linear regression models were adjusted for sex, age, smoking status, and region.

## Data Availability

The data presented in this study are available upon request from the corresponding author. The data are not publicly available because they contain sensitive patient information and location data.

## Supplementary Materials

The following supporting information can be downloaded at: https://www.mdpi.com/article/10.3390/toxics13080703/s1, Table S1. Results of regression analysis on the associations between demographic variables and depression and anxiety Scores.

## Abbreviations

The following abbreviations are used in this manuscript:

PHQ-9	Patient Health Questionnaire-9
GAD-7	Generalized Anxiety Disorder-7
HADS	Hospital Anxiety and Depression Scale
KNHANES	Korea National Health and Nutrition Examination Survey
