# Supplementary material for: Depressive and Anxiety Symptoms Among Patients with Asbestos-Related Diseases in Korea
Source: Toxics. 2025 Aug 21;13(8):703. doi: 10.3390/toxics13080703 (PMC12390261; doi:10.3390/toxics13080703)
Supplement: Supplementary file 1 [file toxics-13-00703-s001.zip › toxics-3796029-supplementary.pdf]

Supplemental Materials

Depressive and Anxiety Symptoms Among Patients with Asbestos-related Diseases in Korea

Min-Sung Kang [1,2], Mee-Ri Lee [3] and Young Hwangbo [3,*]

[1] Asbestos Environmental Health Center, Soonchunhyang University Cheonan Hospital, Soonchunhyang 6-gil 31, Dongnam-gu, Cheonan-si 31151, Republic of Korea; kms83korea03@hanmail.net

[2] Division of Medical Science, Soonchunhyang University, 22, Soonchunhyang-ro, Sinchang-myeon, Asan-si, 31538, Republic of Korea

[3] Department of Preventive Medicine, Soonchunhyang University, College of Medicine, Soonchunhyang 6-gil 31, Dongnam-gu, Cheonan-si 31151, Republic of Korea; meeri@sch.ac.kr

* Correspondence: hbyoung@sch.ac.kr

Table S1. Results of regression analysis on the associations between demographic variables and depression and anxiety Scores.

Variables	PHQ-9		GAD-7		HADS-A		HADS-D	
	β (95% CI)	p	β (95% CI)	p	β (95% CI)	p	β (95% CI)	p
Sex								
Male	0.41 (-1.11, 1.92)	0.599	0.00 (-1.37, 1.36)	0.997	0.46 (-0.86, 1.77)	0.497	0.78 (-0.44, 2.01)	0.209
Female	Ref.		Ref.		Ref.		Ref.	
Age								
50-59	-3.58 (-10.65, 3.50)	0.010	-3.98 (-10.35, 2.39)	0.009	-5.48 (-11.57, 0.61)	0.027	-7.39 (-12.99, -1.78)	0.007
60-69	-0.86 (-4.79, 3.50)		-1.90 (-5.43, 1.64)		-3.66 (-7.04, -0.28)		-4.84 (-7.95, -1.73)	
70-79	-0.01 (-3.63, 3.62)		-1.27 (-4.53, 1.99)		-2.02 (-5.14, 1.10)		-2.74 (-5.61, 0.13)	
80-89	0.45 (-3.19, 4.09)		-0.90 (-4.18, 2.37)		-1.80 (-4.93, 1.33)		-2.67 (-5.56, 0.21)	
≥90	Ref.		Ref.		Ref.		Ref.	
Smoking status								
Never	-2.83 (-4.55, -1.11)	0.046	-2.63 (-4.18, -1.08)	0.043	-2.89 (-4.37, -1.40)	0.058	-1.99 (-3.39, -0.59)	0.031
Past smoker	-2.56 (-4.46, -0.66)		-1.85 (-3.57, -0.14)		-2.07 (-3.71, -0.43)		-1.49 (-3.04, 0.06)	
Current smoker	-2.18 (-6.61, 2.24)		-1.92 (-5.91, 2.07)		-2.89 (-6.71, 0.92)		-0.36 (-3.97, 3.25)	
Unknown	Ref.		Ref.		Ref.		Ref.	
Region								
Seoul	-0.48 (-5.59, 4.62)	0.025	-1.35 (-5.92, 3.23)	0.032	0.02 (-4.34, 4.38)	0.055	-2.31 (-6.44, 1.81)	0.026
Incheon	-0.27 (-5.01, 4.47)		-0.28 (-4.52, 3.97)		1.93 (-2.12, 5.97)		-1.91 (-5.74, 1.92)	
Gyeonggi-do	0.25 (-2.87, 3.37)		-0.85 (-3.64, 1.95)		0.00 (-2.66, 2.67)		-0.66 (-3.18, 1.87)	
Gyeonsangsam-do	-4.65 (-8.85, -0.45)		-2.96 (-6.73, 0.81)		-3.42 (-7.01, 0.17)		-2.93 (-6.32, 0.47)	
Busan	1.21 (-0.71, 3.12)		1.89 (0.17, 3.61)		2.64 (1.00, 4.28)		0.86 (-0.69, 2.41)	
Chungcheongnam-do	Ref.		Ref.		Ref.		Ref.	
